# A Comprehensive Insight Into Primary Intimal Sarcoma of the Pulmonary Artery; From Diagnosis to Management: A Case Report and Review of the Literature

**DOI:** 10.1002/ccr3.9580

**Published:** 2024-11-18

**Authors:** Azin Alizadehasl, Soroush Najdaghi, Maryam Mohseni Salehi, Shahla Meshgi, Seyedeh Fatemeh Hosseini Jebelli, Azam Yalameh Aliabadi, Hoda Hakimian, Sara Forati, Amineh Safavirad, Delaram Narimani Davani

**Affiliations:** ^1^ Cardio‐Oncology Research Center Rajaie Cardiovascular Medical and Research Institute Tehran Iran; ^2^ Heart Failure Research Center, Cardiovascular Research Institute Isfahan University of Medical Science Isfahan Iran; ^3^ Department of Cardiac Imaging, Rajaie Cardiovascular Medical and Research Center, School of Medicine Iran University of Medical Sciences Tehran Iran

**Keywords:** cardiac MRI, case report, chemotherapy, intimal sarcoma, pulmonary artery mass, radiotherapy

## Abstract

Primary intimal sarcoma of the pulmonary artery is a rare and aggressive malignancy that presents significant diagnostic and therapeutic challenges due to its nonspecific symptoms and propensity for late detection. This case report aimed to elucidate the diagnostic journey, surgical intervention, and multidisciplinary management of this rare entity. In September 2023, a 42‐year‐old male presented with dyspnea on exertion and retrosternal chest pain, classified as NYHA FC II. Initial investigations, including ECG and lab tests, indicated tachycardia and elevated troponin and NT‐pro‐BNP levels. Transthoracic and transesophageal echocardiography identified a multilobulated mass in the right ventricular outflow tract and main pulmonary artery. Cardiac MRI and CT angiography confirmed a high‐grade pleomorphic spindle cell tumor, leading to surgical resection in October 2023. Histopathology confirmed intimal sarcoma. Postsurgery, the patient underwent chemotherapy and radiotherapy, showing significant clinical improvement and no recurrence on follow‐up PET‐CT. This case highlights the importance of a multidisciplinary approach in diagnosing and managing primary intimal sarcoma of the pulmonary artery, emphasizing the role of advanced imaging, timely surgical intervention, and combined chemotherapy with radiotherapy in improving patient outcomes.

**Abbreviations**
CK: cytokeratin
CMR: cardiac magnetic resonance
CPB: cardiopulmonary bypass
CPTE: chronic pulmonary thromboembolism
CT: computed tomography
DOE: dyspnea on exertion
EBUS: endobronchial ultrasound
EBUS‐TBNA: endobronchial ultrasound‐guided transbronchial needle aspiration
ECG: electrocardiogram
FDG: fluorodeoxyglucose
IHC: immunohistochemistry
ISPA: intimal sarcoma of the pulmonary artery
LGE: late gadolinium enhancement
LPA: left pulmonary artery
LVEF: left ventricular ejection fraction
MPA: main pulmonary artery
MRI: magnetic resonance imaging
NT‐pro‐BNP: N‐terminal pro B‐type natriuretic peptide
NYHA: New York Heart Association
PA: pulmonary artery
PEA: pulmonary endarterectomy
PET: positron emission tomography
PG: pressure gradient
PS: pulmonic stenosis
PV: pulmonary valve
RPA: right pulmonary artery
RVE: right ventricular enlargement
RVEF: right ventricular ejection fraction
RVOT: right ventricular outflow tract
S4: fourth heart sound
SBRT: stereotactic body radiation therapy
SI: signal intensity
SMA: smooth muscle actin
STIR: short‐tau inversion recovery
STJ: sinotubular junction
T1W: T1‐weighted
T2‐STIR: T2‐weighted short‐tau inversion recovery
TEE: transesophageal echocardiogram
TTE: transthoracic echocardiography
VBG: venous blood gas
VEGFR2: vascular endothelial growth factor receptor 2
WBC: white blood cells

**Summary**

- Primary intimal sarcoma of the pulmonary artery, despite its rarity and diagnostic complexity, can be effectively managed through a multidisciplinary approach.
- Early diagnosis, utilizing advanced imaging techniques, combined with prompt surgical resection and adjuvant chemotherapy and radiotherapy, can significantly improve patient outcomes and reduce recurrence risk.

## 1 Background

Primary intimal sarcoma of the pulmonary artery (ISPA), a rare malignant mesenchymal tumor, challenges the realms of medical comprehension, with a scant 400 documented cases in the literature [1, 2]. Originating from the intimal layer of the vascular wall, this formidable adversary obstructs the lumen, infiltrates the vascular wall, and poses a significant risk of distant metastasis. Its elusive presentation often masquerades as more prevalent pulmonary vascular diseases like chronic pulmonary thromboembolism (CPTE) [1]. Moreover, sophisticated diagnostic tools such as high‐resolution CT scans, color Doppler echocardiography, and molecular testing have revolutionized the identification and differentiation of this rare malignancy [1]. Refined diagnostic criteria, shaped by imaging advancements and molecular insights, guide precise diagnoses. Surgical resection remains the cornerstone of ISPA management, with the extent of resection influencing outcomes [1]. Adjuvant therapies, including radiotherapy and chemotherapy, are areas of ongoing research to complement surgical interventions [3]. The prognosis, while guarded, emphasizes the critical role of early intervention, particularly through surgical resection, in prolonging survival. Here, we present a case report of ISPA and discuss its challenges from diagnosis to treatment.

## 2 Case Presentation

A 42‐year‐old male presented with exertional dyspnea and retrosternal chest pain, classified as New York Heart Association (NYHA) Functional Class II, in September 2023. The symptoms appeared abruptly a month earlier in August 2023 and only occurred during mild activity. The patient had no prior medical history, cardiovascular risk factors, abnormalities in cholesterol levels, smoking status, obesity, or family history of cardiovascular disease. On physical examination, a systolic ejection murmur was heard at the left upper sternal border, along with a diastolic murmur, an accentuated P2, and a right‐sided S4. ECG showed sinus tachycardia. Laboratory results were normal except for mild elevations in troponin (0.2 ng/mL), NT‐pro‐BNP (1350 pg/mL), and venous blood gas (VBG) indicating mixed respiratory and metabolic acidosis (pH = 7.32, pCO_2_ = 5 mmHg, pO_2_ = 35 mmHg, HCO_3_ = 20 mEq/L).

### 2.1 Clinical Evaluation

**FIGURE 1:**
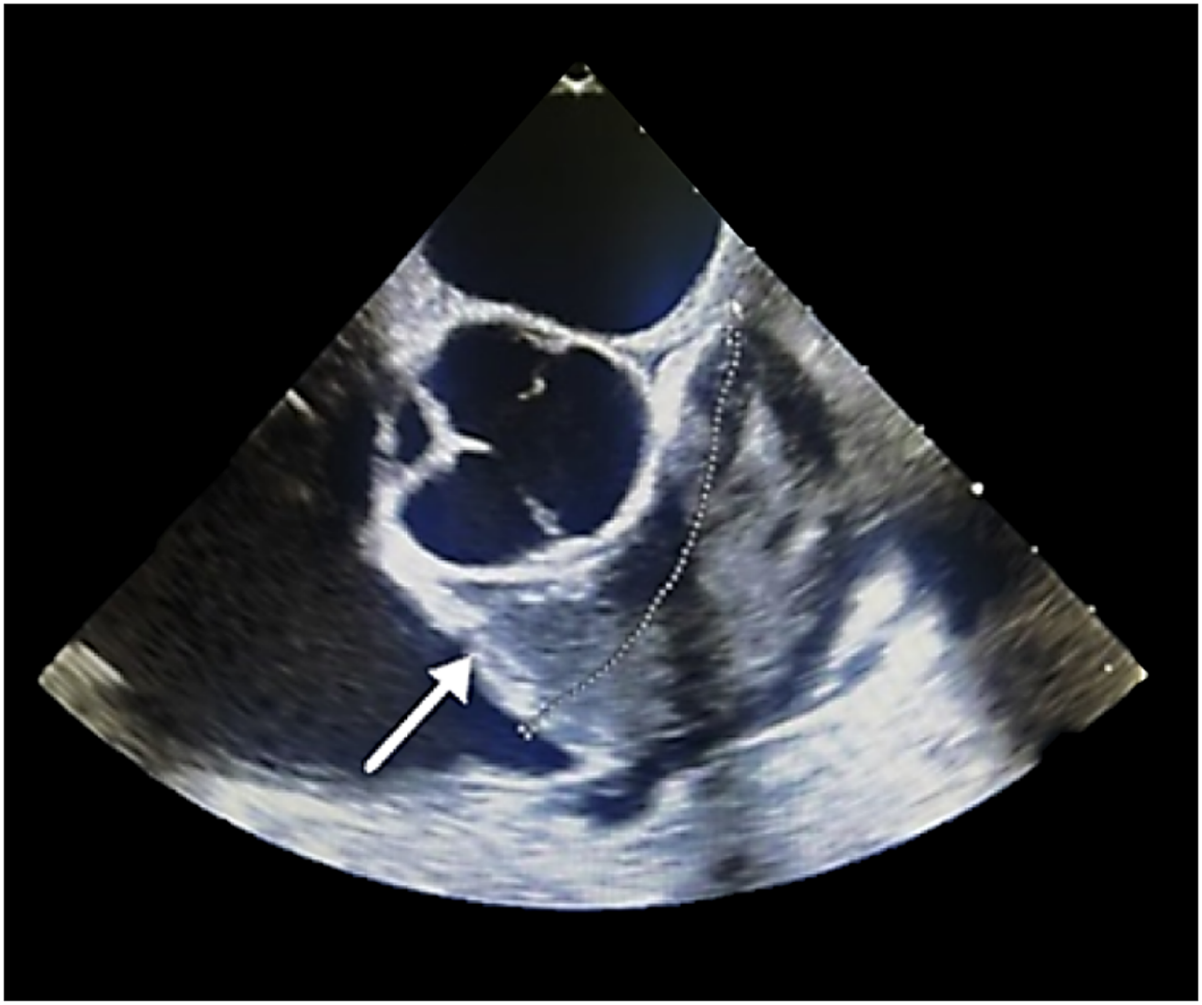
Transesophageal Echocardiogram (short axis view) of a 44‐year‐old patient diagnosed with ISPA. This transesophageal echocardiogram reveals a large, multilobular, broad‐based mass (indicated by the arrow) measuring approximately 5.7 × 2.7 cm. The mass demonstrates tissue‐like echotexture with vascularity, originating from the arterial aspect of the pulmonary valve (PV) leaflet. It extends into both the main pulmonary artery (MPA) and right pulmonary artery (RPA), causing significant systolic turbulence in these regions. This flow disturbance contributes to moderate pulmonary stenosis (PS) with a peak gradient of 59 mmHg. Additionally, the left ventricular ejection fraction (LVEF) is moderately preserved at 55%, while the right ventricular ejection fraction (RVEF) is significantly reduced at 30%, indicative of compromised right ventricular function. ISPA, intimal sarcoma of the pulmonary artery; LVEF, left ventricular ejection fraction; MPA, main pulmonary artery; PS, pulmonic stenosis; PV, pulmonary valve; RPA, right pulmonary artery; RVEF, right ventricular ejection fraction.

The patient was referred to cardiology, where a transthoracic echocardiography (TTE) and transesophageal echocardiography (TEE) revealed a semimobile, multilobulated mass in the right ventricular outflow tract (RVOT), and main pulmonary artery (MPA). The mass extended into the pulmonary valve, causing significant RVOT and pulmonary valve stenosis. Measuring 2.7 × 5.7 cm (axial) and 3.7 cm (coronal), the mass warranted further investigation. The right ventricular ejection fraction (RVEF) was 30%, and the left ventricular ejection fraction (LVEF) was 55% (Figure 1).

### 2.2 Imaging Insights

**FIGURE 2:**
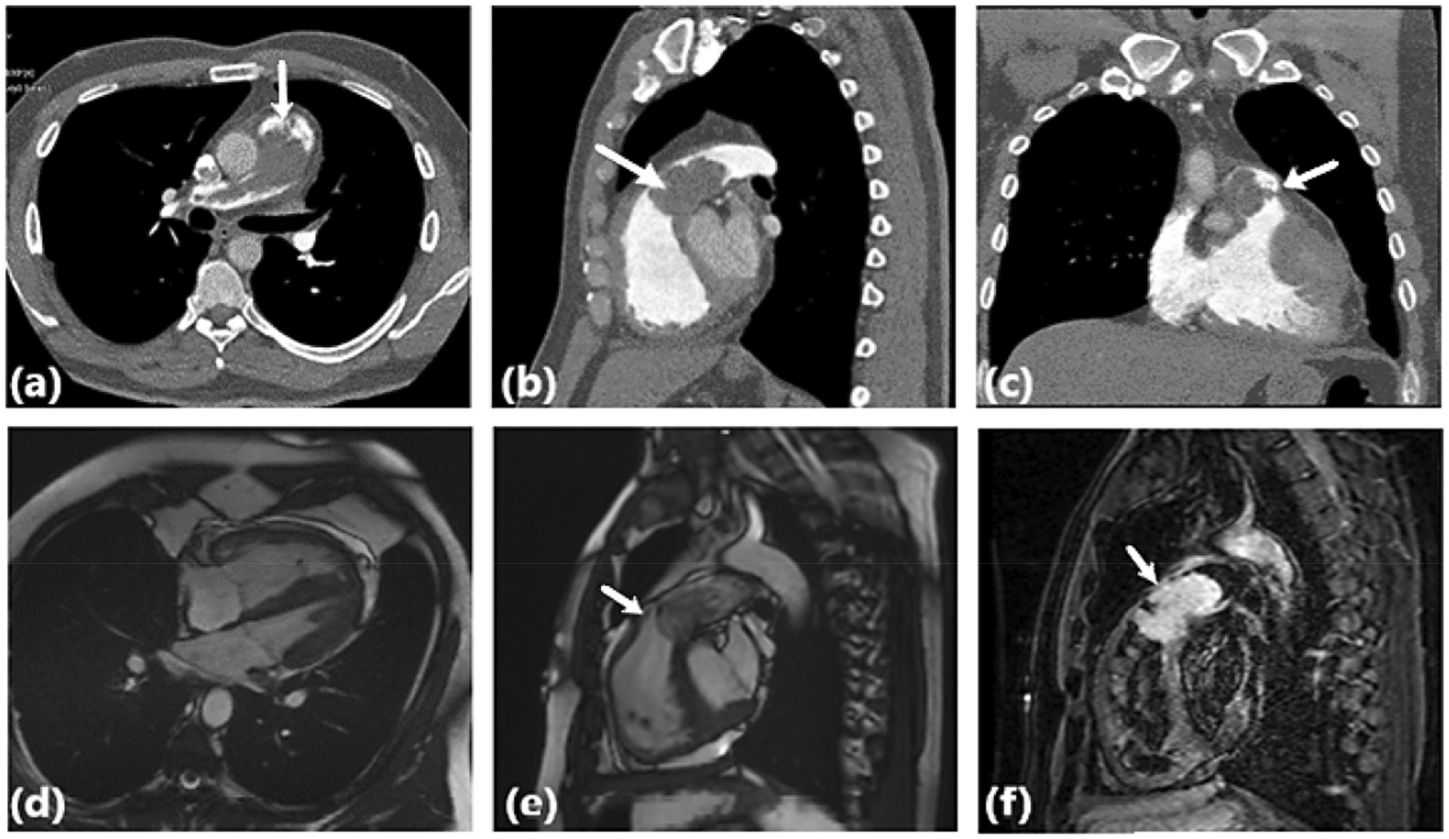
Cardiac CT and MRI Imaging of a 44‐year‐old patient diagnosed with ISPA. CT imaging (axial (a), sagittal (b), coronal (c)) reveals an intraluminal filling defect (6.1 × 3.1 cm) in the right ventricular outflow tract (RVOT), main pulmonary artery (MPA), and extending into the right pulmonary artery (RPA) and right upper lobar branch, involving the pulmonary valve (PV), consistent with intimal sarcoma. The mass causes significant vessel obstruction, potentially leading to pulmonary hypertension and right ventricular (RV) overload. The axial SSFP sequence (c) shows RV enlargement due to pressure overload. The RVOT view (d) reveals a multilobulated mass infiltrating the pulmonary valve, indicating severe RVOT stenosis and mixed tumor composition. On MRI, the lesion is prominently located in the RVOT and extends into the MPA and RPA, including the right upper lobar branch. The mass, approximately 6.1 × 3.1 cm in size, displays a multilobulated structure. T2‐STIR sequences (e) reveal high signal intensity areas, indicating edema and necrosis, while T1‐weighted images (f) highlight viable tumor tissue. The MRI also shows the mass's encasement of the PV, causing severe RVOT stenosis and significant RV enlargement due to pressure overload. CT, computed tomography; ISPA, intimal sarcoma of pulmonary artery; MPA, main pulmonary artery; MRI, magnetic resonance imaging; PV, pulmonary valve; RPA, right pulmonary artery; RV, right ventricle; RVOT, right ventricular outflow tract; SSFP, steady state‐free precession; T1W, T1‐weighted; T2‐STIR, T2 short‐tau inversion recovery.

Multislice spiral computed tomography (CT) angiography with reconstructed views and dynamic contrast medium provided detailed imaging of the mass. It revealed a 30 mm MPA, mild right ventricular enlargement, and an intraluminal filling defect (3.1 × 6.1 cm) extending from the right MPA to the right pulmonary artery (RPA) and right upper lobar branch. The differential diagnosis included a primary pulmonary artery mass such as sarcoma or a metastatic mass (Figure 2a–c).

### 2.3 Cardiac MRI Confirmation

Cardiac MRI (CMR) further characterized the mass, showing isosignal on T1, hypersignal on STIR, and mild vascularity on perfusion sequences. Late gadolinium enhancement (LGE) images displayed scattered enhancements, along with hypointensity areas suggesting thrombosis or necrosis. The mass encased the pulmonary valve, restricting its movement (Figure 2d–f). Given the potential for metastasis, positron emission tomography (PET)‐CT was performed to assess for hypermetabolic lesions elsewhere, but no metastases were detected.

### 2.4 Surgical Intervention and Pathological Evaluation

**FIGURE 3:**
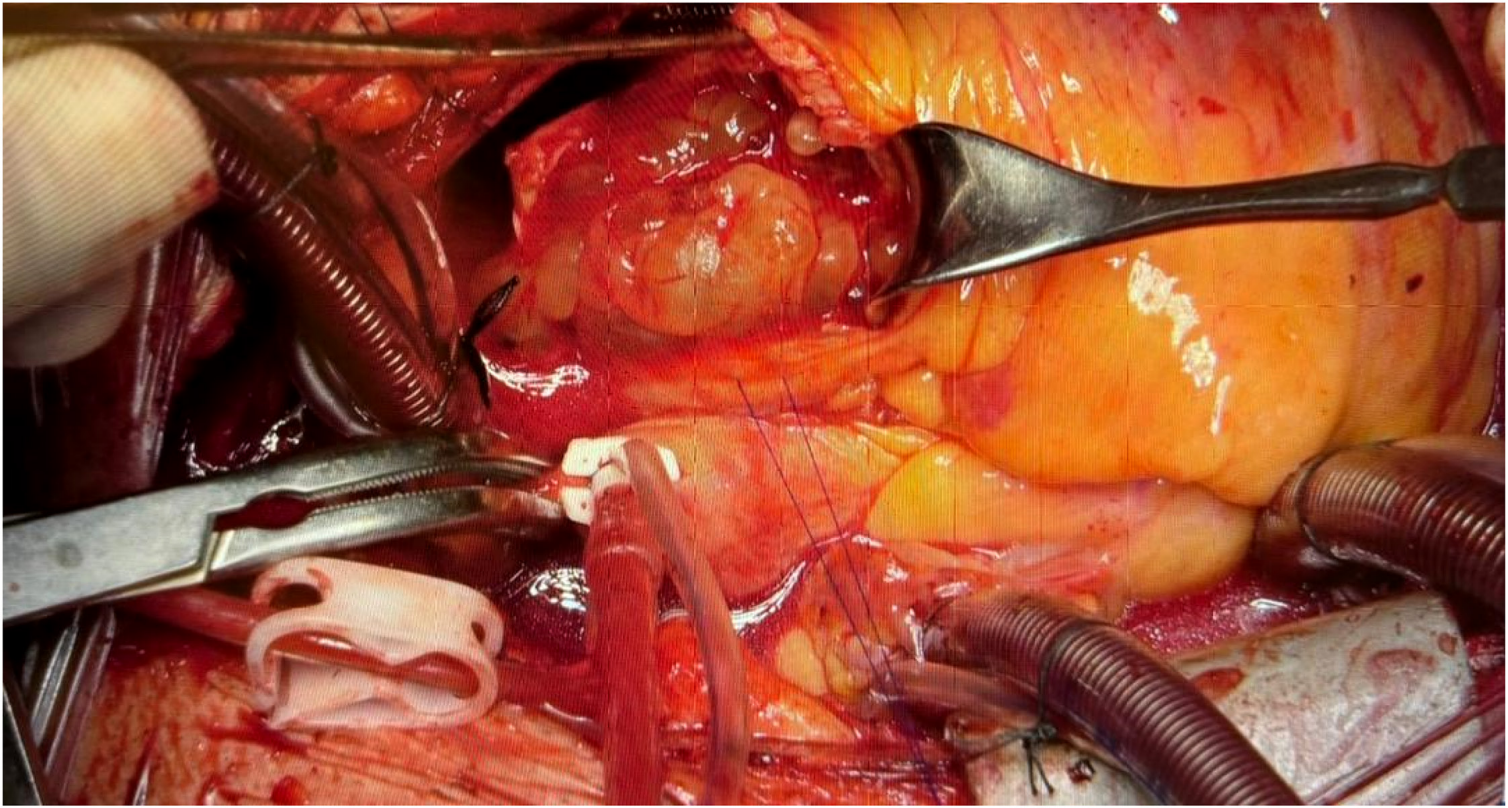
Surgical treatment of a 44‐year‐old patient diagnosed with ISPA. The patient underwent surgery to address a large jelly tumor measuring 3.0 × 6.2 cm. The tumor had an extensive base attached to the pulmonary artery (PA), pulmonary valve (PV), right ventricular outflow tract (RVOT), left pulmonary artery (LPA), and right pulmonary artery (RPA). The aorta was transected above the sinotubular junction (STJ). The RPA was fully opened, and an endarterectomy was performed on the PA, RPA, and LPA. The tumor was nearly completely removed, with a small residual portion penetrating the artery in the upper lobe of the right lung. The RPA was repaired using a pericardial patch, with its branches connected to the RPA and LPA via a pulmonary homograft, and the proximal segment was reattached to the RVOT. ISPA, intimal sarcoma of pulmonary artery; LPA, left pulmonary artery; PA, pulmonary artery; PV, pulmonary valve; RPA, right pulmonary artery; RVOT, right ventricular outflow tract; STJ, sinotubular junction.

Due to the large multilobulated mass in the RVOT and MPA, which extended into the pulmonary valve and caused significant stenosis, surgery was performed in October 2023 to relieve the patient's symptoms of dyspnea and retrosternal chest pain. A large, jelly‐like tumor (3.0 × 6.2 cm), intricately attached to the pulmonary artery, pulmonary valve, RVOT, left pulmonary artery (LPA), and right pulmonary artery (RPA), was excised. Endarterectomy was performed on the pulmonary artery, RPA, and LPA, followed by careful repair and reconstruction (Figure 3).

### 2.5 Histopathological Findings

Histopathological examination of the resected tumor revealed a cellular spindle cell mass with pleomorphic cells, characterized by hyperchromatic oval‐to‐spindle nuclei and poorly defined pink cytoplasm within a myeloid stroma. Immunohistochemical (IHC) staining was negative for CK, S100, CD34, calretinin, SMA, desmin, myogenin, CD31, and ERG markers. However, Ki‐67 staining showed a 50% proliferation rate in tumor cells. These findings confirmed the diagnosis of a high‐grade pleomorphic spindle cell tumor consistent with intimal sarcoma.

### 2.6 Oncological Management and Follow‐Up

Chemotherapy began in January 2023, with the patient receiving four cycles of gemcitabine and docetaxel (Taxotere), administered every 3 weeks. Gemcitabine (1000 mg/m^2^) was given on Days 1 and 8 of each cycle, while docetaxel (75 mg/m^2^) was given on Day 8. This was followed by 30 sessions of radiotherapy from May 1 to June 14, 2023, totaling 60 Gy.

A multidisciplinary team of oncologists, cardiothoracic surgeons, radiologists, and pathologists provided coordinated care throughout the treatment. Regular follow‐ups were conducted to monitor the patient's recovery and manage potential side effects. In November 2023, a PET‐CT scan, conducted 4 months postsurgery and 1 month after chemotherapy, revealed no hypermetabolic lesions in the pulmonary artery or elsewhere, suggesting no recurrence of the disease.

By May 2024, the patient reported significant clinical improvement, with no signs of shortness of breath or chest pain. Follow‐up evaluations confirmed stable cardiovascular function (LVEF 55%, RVEF 45%), normal levels of troponin and NT‐pro‐BNP, and a normal sinus rhythm on ECG, indicating a successful response to the comprehensive oncological treatment.

## 3 Discussion

ISPA is a rare and aggressive malignancy, accounting for only 0.001%–0.003% of tumors, with onset ranging from 2 months to 89 years of age, predominantly affecting females [4, 5]. Due to its nonspecific symptoms and imaging similarities to pulmonary embolism, ISPA is frequently misdiagnosed, delaying accurate diagnosis, and significantly impacting treatment outcomes [2, 5]. This section explores innovative treatment approaches and the challenges in managing ISPA.

### 3.1 Surgical Interventions

Surgical resection of ISPA, combined with pulmonary endarterectomy (PEA) via cardiopulmonary bypass and deep hypothermic circulatory arrest, has been shown to maximize tumor removal in nonmetastatic cases. Despite aggressive surgical measures, the average survival time postsurgery is around 11.6 months, with a high risk of recurrence [6]. However, studies suggest that more extensive surgical resections, including right pneumonectomy and reconstruction, can prevent relapse and improve survival rates, with one case showing disease‐free survival 16 months postsurgery [7].

### 3.2 Chemotherapy

Traditional chemotherapy regimens, particularly those based on doxorubicin, have shown poor outcomes for ISPA patients. A 2008 study by Penel et al. [8] revealed a median survival of only 8 months among eight patients treated with doxorubicin. Similarly, a 2023 meta‐analysis by Tanaka et al. [9] confirmed that while doxorubicin remains a standard treatment for soft tissue sarcomas, combination therapies have not significantly improved survival. Alternative chemotherapy regimens, such as ifosfamide and epirubicin, have shown tumor regression in select cases, though side effects often limit their use. Newer combinations, such as vinorelbine and cisplatin, have demonstrated promising results in heavily pretreated patients [1].

In our case, we employed a combination of gemcitabine and docetaxel (Taxotere), which resulted in significant clinical and imaging improvement 4 months postchemotherapy. However, due to short‐term follow‐up, long‐term efficacy remains uncertain.

### 3.3 Adjuvant and Neoadjuvant Radiotherapy

While surgery remains the cornerstone of ISPA treatment, adjuvant radiotherapy has shown promise in controlling tumor progression. For example, a case study by Allen et al. highlighted the use of neoadjuvant radiotherapy to reduce tumor size before surgery, enabling successful tumor removal and disease control [3]. Similarly, Romanowska et al. reported that adjuvant radiotherapy effectively controlled local disease postresection, with no recurrence [10]. In our case, the patient underwent 30 sessions of radiotherapy, delivering a total of 60 Gy, and subsequent PET‐CT scans revealed no hypermetabolic lesions, suggesting successful disease control.

Table 1 provides a survey of various treatment approaches and their outcomes, offering insights into the evolving management of ISPA.

**TABLE 1:** Summary of treatment options with their case implication for ISPA.

Treatment option	Case implication
Surgical resection [1, 4, 6]	Complete surgical resection with pulmonary endarterectomy (PEA) is the primary treatment. It may involve extensive reconstructive measures and cardiopulmonary bypass.
Neoadjuvant radiation [3]	Preoperative radiotherapy to improve resectability in cases of borderline resectable tumors.
Adjuvant chemotherapy [1]	Chemotherapy after surgery to reduce the risk of recurrence. Drugs used include doxorubicin, cisplatin, and ifosfamide.
Pazopanib therapy [1]	Use of the tyrosine kinase inhibitor pazopanib for unresectable or recurrent tumors.
Radiotherapy [1]	Postoperative radiotherapy to control local recurrence.
Combination chemotherapy [1]	Use of combination chemotherapy regimens like ifosfamide and epirubicin, or vinorelbine and cisplatin for inoperable or recurrent cases.
Targeted therapy with apatinib [11]	Use of the VEGFR2 inhibitor apatinib for recurrent cases.
Stereotactic body radiation Therapy (SBRT) [12]	Use of SBRT for treating lung metastases and delaying palliative chemotherapy.
Chemotherapy with gemcitabine and docetaxel [3]	Postoperative use of gemcitabine and docetaxel for recurrent cases.
Ex vivo lung autotransplantation [13]	Use of lung autotransplant techniques for the treatment of pulmonary artery sarcoma.

Abbreviations: CPB: cardiopulmonary bypass; PEA: pulmonary endarterectomy; SBRT: stereotactic body radiation therapy; TKI: tyrosine kinase inhibitor; VEGFR2: vascular endothelial growth factor receptor 2.

### 3.4 Diagnostic Challenges and Misdiagnosis

Accurate diagnosis of intimal sarcoma of the pulmonary artery (ISPA) requires a multimodal imaging approach, utilizing CT, MRI, PET, echocardiography, and endobronchial ultrasound‐guided needle aspiration (EBUS‐NA). Each imaging modality offers distinct advantages, helping to differentiate ISPA from conditions like pulmonary thromboembolism, ultimately guiding appropriate treatment, and improving prognosis [1]. Table 2 includes multiple imaging modalities with their insight through ISPA diagnosis.

**TABLE 2:** Different imaging findings and their features in the diagnosis of ISPA.

Imaging insight	Description
CT [14, 15]	Identifies ISPA through features like tumoral impaction, heterogeneous attenuation, and intratumoral vessels. 3D reconstruction helps differentiate ISPA from thromboembolism.
MRI [15, 16]	Provides high spatial resolution and functional assessment. Shows inhomogeneous delayed enhancement, suggesting intermingled thrombi.
PET [17, 18]	FDG‐PET shows hypermetabolism, distinguishing ISPA from thromboembolism. Low cellularity ISPA may show poor FDG uptake.
Echocardiography [14]	Reveals mass‐like lesions in the pulmonary artery, aiding in diagnosis when combined with other imaging modalities.
EBUS [19]	EBUS‐TBNA provides real‐time imaging of lymph nodes and pulmonary arteries, useful for obtaining tissue samples and definitive preoperative diagnosis.

Abbreviations: CT: computed tomography; EBUS: endobronchial ultrasound; EBUS‐TBNA: endobronchial ultrasound‐guided transbronchial needle aspiration; FDG: fluorodeoxyglucose; MRI: magnetic resonance imaging; ISPA: intimal sarcoma of pulmonary artery; PET: positron emission tomography.

The definitive diagnosis is based on histopathological examination following surgical resection. Immunohistochemistry (IHC) is essential, with ISPA tumors often testing positive for markers such as vimentin, smooth muscle actin (SMA), and MDM2. However, differentiating ISPA from other sarcomas, such as leiomyosarcoma, can be challenging in the absence of typical morphologic features [1]. In our case, IHC staining of the surgical sample, including Ki‐67 proliferation activity, confirmed the diagnosis of high‐grade pleomorphic spindle cell intimal sarcoma. Key histological features of ISPA are summarized in Table 3.

**TABLE 3:** Histological findings of ISPA with their features description.

Histological features	Description
Sclerosing extracellular matrix [20]	Sclerosing appearance with few spindle/pleomorphic cells, often confused with chronic thromboembolic pulmonary hypertension.
Spindle and pleomorphic cells [20, 21]	The proliferation of atypical spindle and pleomorphic cells within a myxomatous matrix.
Myxoid background [21]	Presence of myxoid matrix in the histological examination.
MDM2 expression [20, 21]	Intense MDM2 expression was observed in tumor cells.
Genetic alterations [21]	Frequent gains and amplifications in the 12q13‐14 region.
Immunohistochemical markers [21]	Strong vimentin expression; focal positivity for alpha‐smooth muscle Actin, CD117, CD68, p53, and bcl2; negative for endothelial markers.
Proliferation index (Ki‐67) [21]	Variable Ki‐67 index ranging from 5% to 80%.
Intimal thickening [22]	Tumor causes arteriosclerosis‐like intimal thickening extending from the main to subsegmental arteries.
Eccentric intimal thickening or recanalization [22]	Observed in distal small arteries due to peripheral tumor emboli or pulmonary hypertension induced by the proximal tumor.
Necrotizing background with low cellularity [18]	Some ISPA cases show low cellularity with a necrotizing background, leading to poor FDG uptake in PET/CT scans.
Cytologic features from EBUS‐TBNA [19, 23]	Pleomorphic malignant spindled cells arranged in loosely cohesive clusters are useful for diagnosis with ancillary studies like immunohistochemistry.

Abbreviations: CT: computed tomography; EBUS‐TBNA: endobronchial ultrasound‐guided transbronchial needle aspiration; FDG: fluorodeoxyglucose; ISPA: intimal sarcoma of pulmonary artery; PET: positron emission tomography.

### 3.5 Innovative Approaches and Future Directions

Newer targeted therapies, such as apatinib, have shown potential in stabilizing ISPA postsurgery, presenting a promising avenue for future treatment development. In a 2020 case report by Lu & Yin et al. (11), a 44‐year‐old male experienced multiple lesions in the pulmonary trunk, left pulmonary artery, mediastinum, and pericardium, indicating recurrence after tumor resection. Although the patient tolerated apatinib with manageable side effects, he passed away 19 months postsurgery. This case highlights the need for larger clinical trials to establish the safety and efficacy of apatinib for ISPA on a broader scale.

In addition, innovative surgical techniques have been introduced to improve outcomes in ISPA treatment. For instance, Obeso Carillo et al. [24] in 2015 proposed using self‐made stapled bovine pericardial graft conduits for pulmonary artery reconstruction following tumor resection. This method enhances surgical safety and effectiveness by eliminating the need for long‐term anticoagulation and providing excellent biocompatibility and graft durability. However, this technique is technically demanding, requiring advanced surgical expertise. Additionally, it carries the risk of conduit‐related complications, such as stenosis or structural failure, making precise intraoperative management essential for optimal results.

## 4 Conclusion

We presented a patient with ISPA, referred with dyspnea and retrosternal chest pain. Surgical resection followed by chemotherapy (gemcitabine and docetaxel) and radiotherapy, achieved favorable outcomes with no evidence of disease recurrence on follow‐up PET‐CT, underscoring the significance of comprehensive oncological strategies in managing rare malignancies effectively.

## Author Contributions

**Azin Alizadehasl:** supervision, writing – review and editing. **Soroush Najdaghi:** conceptualization, resources, software, visualization. **Maryam Mohseni Salehi:** methodology, project administration, validation. **Shahla Meshgi:** data curation, investigation. **Seyedeh Fatemeh Hosseini Jebelli:** resources, validation, writing – original draft. **Azam Yalameh Aliabadi:** methodology, project administration, writing – original draft. **Hoda Hakimian:** formal analysis, software, writing – review and editing. **Sara Forati:** conceptualization, methodology, validation, visualization. **Amineh Safavirad:** software, writing – review and editing. **Delaram Narimani Davani:** writing – original draft, writing – review and editing.

## Ethics Statement

Informed consent was obtained from the patient for participation and publication of this case report.

## Consent

Written informed consent was obtained from the patient for the publication of this case report and any accompanying images. All identifying information was removed to ensure privacy. A copy of the written consent is available for review by the Editor of this journal.

## Conflicts of Interest

The authors declare no conflicts of interest.

## Data Availability

All data generated or analyzed during this study are included in this published article.

## References

[1] T. Assi , J. Kattan , E. Rassy , et al., “A Comprehensive Review on the Diagnosis and Management of Intimal Sarcoma of the Pulmonary Artery,” Critical Reviews in Oncology/Hematology 147 (2020): 102889. 32035299 10.1016/j.critrevonc.2020.102889

[2] A. Mahdi , M. Mahdi , and P. J. C. Ters , “A Journey From Cardiology to Oncology Reveals a Rare Case of Primary Intimal Sarcoma in a Patient With Dyspnea: A Case Report,” Cureus 15 (2023): e38439. 37273385 10.7759/cureus.38439 PMC10234616

[3] A. J. Allen , S. C. Smith , R. Pillappa , et al., “Intimal Sarcoma of the Pulmonary Artery Treated With Neoadjuvant Radiation Prior to Pulmonary Artery Resection and Reconstruction,” Respiratory Medicine Case Reports 33 (2021): 33. 10.1016/j.rmcr.2021.101414 PMC8348529 34401262

[4] D.‐Y. Chang , K.‐C. Lin , J. Y. Pan , H.‐W. Liu , S.‐H. Kuo , and L. L. T. J. R. C. R. Lee , “Pulmonary Artery Intimal Sarcoma: A Case Report and Literature Review,” Respirology Case Reports 8 (2020): e00530. 32042432 10.1002/rcr2.530 PMC7002898

[5] J. Van Dievel , R. Sciot , M. Delcroix , et al., “Single‐Center Experience With Intimal Sarcoma, an Ultra‐Orphan, Commonly Fatal Mesenchymal Malignancy,” Oncology Research and Treatment 40 (2017): 353–359. 28501860 10.1159/000476036

[6] Y. Han , Y. Zhen , X. Liu , et al., “Surgical Treatment of Primary Pulmonary Artery Sarcoma. General Thoracic and Cardiovascular,” Surgery 69 (2020): 638–645. 10.1007/s11748-020-01476-2 PMC7981312 32918676

[7] Y. Yamamoto , Y. Shintani , S. Funaki , et al., “Aggressive Surgical Resection of Pulmonary Artery Intimal Sarcoma,” Annals of Thoracic Surgery 106, no. 4 (2018): e197–e199. 29730353 10.1016/j.athoracsur.2018.03.072

[8] N. Penel , S. Taieb , L. Ceugnart , et al., “Report of Eight Recent Cases of Locally Advanced Primary Pulmonary Artery Sarcomas: Failure of Doxorubicin‐Based Chemotherapy,” Journal of Thoracic Oncology 3 (2008): 907–911. 18670310 10.1097/JTO.0b013e318180720d

[9] K. Tanaka , M. Kawano , T. Iwasaki , I. Itonaga , and H. Tsumura , “A Meta‐Analysis of Randomized Controlled Trials That Compare Standard Doxorubicin With Other First‐Line Chemotherapies for Advanced/Metastatic Soft Tissue Sarcomas,” PLoS One 14 (2019): e0210671. 30629708 10.1371/journal.pone.0210671 PMC6328231

[10] A. B. Romanowska , E. Lewicka , G. Sławiński , H. Jankowska , and R. Zaucha , “Case Report: Adjuvant Radiotherapy Can be an Effective Treatment for Intimal Sarcoma of the Heart. Frontiers,” Oncology 11 (2021): 11. 10.3389/fonc.2021.621289 PMC7953907 33718180

[11] P. Lu and B. Yin , “Misdiagnosis of Primary Intimal Sarcoma of the Pulmonary Artery as Chronic Pulmonary Embolism: A Case Report,” World Journal of Clinical Cases 8, no. 5 (2020): 986–994. 32190637 10.12998/wjcc.v8.i5.986 PMC7062609

[12] S. García‐Cabezas , M. Centeno‐Haro , S. Espejo‐Pérez , et al., “Intimal Sarcoma of the Pulmonary Artery With Multiple Lung Metastases: Long‐Term Survival Case,” World Journal of Clinical Oncology 8, no. 4 (2017): 366–370. 28848704 10.5306/wjco.v8.i4.366 PMC5554881

[13] D. Nakajima , S. Miyahara , and H. Date , “Reconstruction Surgery Case Report: Ex Vivo Surgery and Auto Lung Transplantation for Pulmonary Artery Sarcoma,” Journal of Visceral Surgery 6 (2020): 2.

[14] E.‐Y. Choi , Y. W. Yoon , H. M. Kwon , et al., “A Case of Pulmonary Artery Intimal Sarcoma Diagnosed With Multislice CT Scan With 3D Reconstruction,” Yonsei Medical Journal 45, no. 3 (2004): 547–551. 15227746 10.3349/ymj.2004.45.3.547

[15] C. Kim , M. Y. Kim , J.‐W. Kang , J. S. Song , K. Y. Lee , and S.‐S. Kim , “Pulmonary Artery Intimal Sarcoma Versus Pulmonary Artery Thromboembolism: CT and Clinical Findings,” Korean Journal of Radiology 19 (2018): 792–802. 29962886 10.3348/kjr.2018.19.4.792 PMC6005959

[16] M. Liu , Z.‐h. Ma , T. Jiang , et al., “Differential Diagnosis of Pulmonary Artery Sarcoma and Central Chronic Pulmonary Thromboembolism Using CT and MR Images,” Heart, Lung & Circulation 27, no. 7 (2017): 819–827. 10.1016/j.hlc.2017.06.716 29032917

[17] D.‐H. Lee , T.‐E. Jung , J. H. Lee , D.‐g. Shin , W.‐J. Park , and J. H. Choi , “Pulmonary Artery Intimal Sarcoma: Poor 18F‐Fluorodeoxyglucose Uptake in Positron Emission Computed Tomography,” Journal of Cardiothoracic Surgery 8 (2013): 40. 23497592 10.1186/1749-8090-8-40 PMC3605187

[18] T. Takauchi , R. Murai , K. Musiake , et al., “Pedunculated Pulmonary Artery Intimal Sarcoma With Poor Uptake in 18F‐FDG PET/CT: A Case Report,” Journal of Cardiology Cases 24, no. 3 (2021): 110–113. 34466172 10.1016/j.jccase.2021.02.006 PMC8380851

[19] N. P. Caraway , D. Salina , M. T. Deavers , R. C. Morice , and G. Landon , “Pulmonary Artery Intimal Sarcoma Diagnosed Using Endobronchial Ultrasound‐Guided Transbronchial Needle Aspiration,” CytoJournal 12 (2015): 3. 25745502 10.4103/1742-6413.151667 PMC4345650

[20] K. Amemiya , M. Nishihira , H. Ishibashi‐Ueda , et al., “A 5‐Year Survivor of Endarterectomy for Sclerosing Undifferentiated Intimal Sarcoma of the Pulmonary Artery: Importance of Clinical Suspicion and Careful Histologic Evaluation,” Pulmonary Circulation 13, no. 4 (2023): e12315. 38034856 10.1002/pul2.12315 PMC10687324

[21] B. Bode‐Lesniewska , J. Zhao , E. Speel , et al., “Gains of 12q13–14 and Overexpression of mdm2 Are Frequent Findings in Intimal Sarcomas of the Pulmonary Artery,” Virchows Archiv 438 (2001): 57–65. 11213836 10.1007/s004280000313

[22] A. Ro , S. Mori , H. Sugiura , et al., “An Autopsy Case of Pulmonary Artery Intimal Sarcoma: Detailed Observation of Tumor and Its Related Lesions in Pulmonary Arteries,” Cardiovascular Pathology: The Official Journal of the Society for Cardiovascular Pathology 43 (2019): 107143. 31437715 10.1016/j.carpath.2019.07.002

[23] A. Harbhajanka , W. Dahoud , C. W. Michael , and R. Elliot , “Cytohistological Correlation, Immunohistochemistry and Murine Double Minute Clone 2 Amplification of Pulmonary Artery Intimal Sarcoma: A Case Report With Review of Literature,” Diagnostic Cytopathology 47 (2018): 494–497. 30552756 10.1002/dc.24131

[24] G. A. Obeso Carillo , R. Casais Pampín , J. J. Legarra Calderón , and M. G. Pradas , “Primary Pulmonary Artery Sarcoma: A New Surgical Technique for Pulmonary Artery Reconstruction Using a Self‐Made Stapled Bovine Pericardial Graft Conduit,” European Journal of Cardio‐Thoracic Surgery: Official Journal of the European Association for Cardio‐Thoracic Surgery 47, no. 1 (2015): 188–190. 25000937 10.1093/ejcts/ezu269

